# The efficacy and safety of acupoint catgut embedding for peripheral facial paralysis: A protocol for systematic review and meta analysis

**DOI:** 10.1097/MD.0000000000027680

**Published:** 2021-11-19

**Authors:** Fengyi Wang, Qinglin Li, Qiaoyun Yu, Junquan Liang, Yunxiang Xu, Guizhen Chen

**Affiliations:** a The Bao‘an District TCM Hospital, The Affiliated Hospital of Guangzhou University of Chinese Medicine, Guangzhou University of Chinese Medicine, Shenzhen, Guangdong, China; b Clinical Medical School of Acupuncture, Moxibustion and Rehabilitation, Guangzhou University of Chinese Medicine, Guangzhou, Guangdong, China.

**Keywords:** acupoint catgut embedding, meta-analysis, peripheral facial paralysis, protocol

## Abstract

**Background:**

Peripheral facial paralysis (PFP) is a consequence of the peripheral neuronal lesion of the facial nerve. It can be either primary (Bell palsy) or secondary. The incidence of PFP is 11.5 to 40.2 per 100,000 people a year. Nearly 70% of patients with PFP recover completely, but almost 30% of patients leave multiple sequelae which caused impacts on the patient's quality of life, both physically and psychologically. The conventional treatments of PFP are limited for some person because of side-effects. Previous studies have suggested that using acupoint catgut embedding (ACE) alone or combined with other therapeutic methods is effective for PFP. However, whether ACE is effective for PFP is still unknown. The purpose of this systematic review (SR) and meta-analysis will summarize the present evidence of ACE used as an intervention for PFP.

**Method/Design:**

Randomized controlled clinical trials that use ACE for PFP will be searched from four international electronic databases (PubMed, Cochrane Library, EMBASE, and Web of Science) and 4 Chinese electronic databases (China National Knowledge Infrastructure, VIP, Wanfang, and China Biology Medicine) to search for relevant literature. We only include studies that were published from the initiation to May 2021. The primary outcomes include effectiveness rate based on House-Brackmann Facial Nerve Grading System. Secondary outcomes will include Sunnybrook facial nerve grading system, Portmann score, facial nerve conduction velocity, Facial Disability Index Scale, adverse events. Two reviewers will perform study selection, data extraction, data synthesis, and quality assessment independently. Assessment of risk of bias and data synthesis will be conducted by using Review Manager 5.3 software. Grade system will be used to evaluate the quality of evidence.

**Discussion:**

This systematic review will help establish clinical evidence regarding the efficacy and safety of acupoint catgut embedding for peripheral facial paralysis.

**Trial registration number:**

CRD42021243212 (PROSPERO)

## 1 Introduction

Peripheral facial paralysis (PFP), also known as idiopathic facial nerve paralysis or Bell palsy, is the most common cranial nerve paralysis. The incidence rate of Bell palsy is 20 to 40 of 100,000 per year, nearly 85% of patients with PFP recover completely, but the rest of patients leave sequelae.^[1,2]^ The common sequelae are incomplete eye closure, crocodile tears, and oral dysfunction during eating, dysphonia, muscle contractures, facial joint movements, and pain. PFP and its sequelae harm their quality of life, both physically and psychologically. The inability to fully express emotions and facial esthetics disorders will lead to the deprivation of social functions of patients.^[3]^ The common treatments of FPF are corticosteroids, antiviral treatment, and surgery. However, corticosteroids are limited for some person because of side-effects and antiviral treatment is uncertainty still. Surgery treatment is expensive and sometimes surgical complications can be caused by it.^[4–6]^

In the theory of traditional Chinese Medicine (TCM), there is no direct disease named PFP. According to the clinical symptoms, PFP belongs to the TCM category of “Koupi,” “Kouwo,” “Diaoxianfeng,” “wry eye and mouth,” and “Intractable Facial Paralysis.” In the theory of TCM, “*pathogenic wind offend face*” is the most common pathogenesis of PFP. Nowadays, peripheral facial paralysis is one of the 64 acupuncture indications recognized by the World Health Organization for its simple and effective even though its mechanism is unclear for modern medicine.^[7]^ Acupuncture therapy has been proven its clinical worth in the treatment of PFP with randomized controlled trials and meta-analysis. In acute stage and sequela stage of Bell palsy, acupuncture therapy can improve facial nerve function and stiffness may be by stimulating nerve fibers in the skin and muscle. Acupuncture appears to influence facial nerve function positively even at long periods after the onset of Bell palsy.^[8–10]^

As a kind of acupuncture external therapy, ACE means implanting lines (eg, catgut suture, protein suture, absorbable sutures) under the skin of acupoints and continuously stimulating the meridians and acupoints. ACE combines TCM acupuncture theory (based on meridians and acupoints) and modern technologies (catgut embedding with a special syringe). ACE has the advantages of frequency, intensity, duration of acupuncture effect than that of conventional acupuncture. Thus, ACE is used in China for intensive treatment in chronic and intractable diseases, PFP is included.^[11]^ However, there is no study on the efficacy and safety of ACE for PFP has been conducted. Our study aims to identify the clinical efficacy and safety of ACE for the treatment of PFP and the benefits or harm it may bring to PFP patients.

## 2 Materials and methods

### 2.1 Study registration

The study protocol has been registered in PROSPERO (registration number: CRD42021243212). The review reporting will be conducted in compliance with the preferred reporting items for systematic reviews and meta-analyses statement guidelines.

### 2.2 Study design

#### 2.2.1 Type of participants

Participants were diagnosed with PFP by clinical doctors according to the diagnostic criteria in the original study. There will be no restriction on sex, race, course, ethnicity, or nation.

Eligibility criteria: As a clinical diagnosis, the characteristics of PFP are acute onset of unilateral lower motor neuron facial paralysis that affects muscles of the upper and the lower face. Emergency symptoms are frequently accompanied by neck, oropharyngeal, or facial numbness, mastoid or ear pain, hyperacusis or altered facial sensation, and disturbed taste on the anterior part of the tongue. Sequelae are incomplete eye closure, crocodile tears, and oral dysfunction during eating, dysphonia, muscle contractures, facial joint movements, and pain.

#### 2.2.2 Type of interventions

All randomized controlled clinical trials (RCTs) reporting the application of ACE for PFP will be included. Patients in the treatment group will be given ACE whether combined with treatments received in the control group, whereas patients in the control group will be given no ACE treatment. The dosages and courses are not limited in our studies.

#### 2.2.3 Types of outcome measures

The primary outcomes will include the effectiveness rate, House-Brackmann Facial Nerve Grading System. Secondary outcomes will include Sunnybrook Facial Nerve Grading System, Portmann Score, Facial Disability Index Scale, and adverse effects.

#### 2.2.4 Inclusion and exclusion criteria

Studies that met all of the following requirements will be included:

1. RCTs comparing ACE with other treatments;
2. Participants were diagnosed with PFP by clinical doctors according to the diagnostic criteria in the original study;
3. The experimental group (ACE group) received ACE whether combined with treatments received in the control group. Besides, for multiple reports of the same research, we only included the latest report.

Studies that met one of the following requirements will be excluded:

1. The diagnostic criteria of the original study did not meet with the clinical diagnosis of PFP;
2. Studies without consistent diagnostic criteria or relevant outcome indicators;
3. Non-English or Chinese-language articles;
4. Duplicate reports or the data cannot be extracted;
5. Case reports, animal experiences, qualitative studies, comments, or review articles.

### 2.3 Literature search strategy

We will comprehensively search four international electronic databases (PubMed, Cochrane Library, EMBASE, and Web of Science) and 4 Chinese electronic databases (CNKI, VIP, Wanfang, and China Biology Medicine) to search for relevant literature. To avoid missing ongoing clinical trials, we will search the following 2 trial registration centers to identify relevant studies: China Clinical Trial Registry (www.chictr.org.cn/index.aspx), The US National Institutes of Health Ongoing Trials Register (www.clinicaltrials.gov), The World Health Organization International Clinical Trials Registry Platform (www.who.int/trialsearch). We only include studies that were published from the initiation to May 2021. These studies must be published in English or Chinese. The literature search will be constructed around search terms for peripheral facial paralysis, search terms for acupoint catgut embedding, search terms for randomized controlled trials, and adapted for each database as necessary. The references of the included studies will also be screened for further material for inclusion. The detailed search strategy for PubMed is in Table 1. Search strategies will also be used for any other electronic databases.

**Table 1:** Search strategy in PubMed.

Serial number	Search items
#1	Facial paralysis[Mesh]
#2	Peripheral facial paralysis sequela[Title/Abstract]
#3	Paralyses, facial[Title/Abstract]
#4	Paralysis, facial[Title/Abstract]
#5	Facial palsy[Title/Abstract]
#5	Facial palsies[Title/Abstract]
#6	Palsies, facial[Title/Abstract]
#7	Palsy, facial[Title/Abstract]
#8	Hemifacial paralysis[Title/Abstract]
#9	Paralyses, hemifacial[Title/Abstract]
#10	Paralysis, hemifacial[Title/Abstract]
#11	Facial paresis[Title/Abstract]
#12	Pareses, facial[Title/Abstract]
#13	Paresis, facial[Title/Abstract]
#14	Facial palsy, lower motor neuron[Title/Abstract]
#15	Lower motor neuron facial palsy[Title/Abstract]
#16	Facial paralysis, peripheral[Title/Abstract]
#17	Facial paralyses, peripheral[Title/Abstract]
#18	Paralysis, peripheral facial[Title/Abstract]
#19	Peripheral facial paralysis[Title/Abstract]
#20	Bell palsy[Mesh]
#21	Bell's palsy[Title/Abstract]
#22	Bell's palsies[Title/Abstract]
#23	Bells palsy[Title/Abstract]
#24	Palsies, Bell's[Title/Abstract]
#25	Palsy, Bell's[Title/Abstract]
#26	Herpetic facial paralysis[Title/Abstract]
#27	Facial paralyses, herpetic[Title/Abstract]
#28	Facial paralysis, herpetic[Title/Abstract]
#29	Herpetic facial paralyses[Title/Abstract]
#30	Paralyses, herpetic facial[Title/Abstract]
#31	Paralysis, herpetic facial[Title/Abstract]
#32	Idiopathic facial nerve paralysis[Title/Abstract]
#33	Facial nerve paralysis, idiopathic[Title/Abstract]
#34	#1 or #2 - #33
#35	Acupoints embedding[Title/Abstract]
#36	Acupoints catgut embedding[Title/Abstract]
#37	Acupuncture points[Title/Abstract]
#38	Acupoints embedding[Title/Abstract]
#39	Acupoints catgut embedding[Title/Abstract]
#40	Acupoints embedding therapy[Title/Abstract]
#42	Acupoints embedding treatment[Title/Abstract]
#43	Acupoints catgut embedding therapy[Title/Abstract]
#44	Acupoints catgut embedding treatment[Title/Abstract]
#45	Acupoint embedding[Title/Abstract]
#46	Acupoint catgut embedding[Title/Abstract]
#47	Acupoint embedding therapy[Title/Abstract]
#48	Acupoint embedding treatment[Title/Abstract]
#49	Acupoint catgut embedding therapy[Title/Abstract]
#50	Acupoint catgut embedding treatment[Title/Abstract]
#51	#35 or #36 - #48
#52	Randomized controlled trial[Publication Type]
#53	Controlled clinical trial[Title/Abstract]
#54	Randomized[Title/Abstract]
#55	Randomly[Title/Abstract]
#56	RCT [Title/Abstract]
#57	#52 or #53 - #56
#58	#34 and #51 and #56

### 2.4 Study selection

As a first step in the data handling process, titles and abstracts of all studies retrieved by the search strategies will be screened for relevance, and all those that are clearly irrelevant will be discarded. If the result is not clearly irrelevant, the full text will be downloaded.

**Figure 1:**
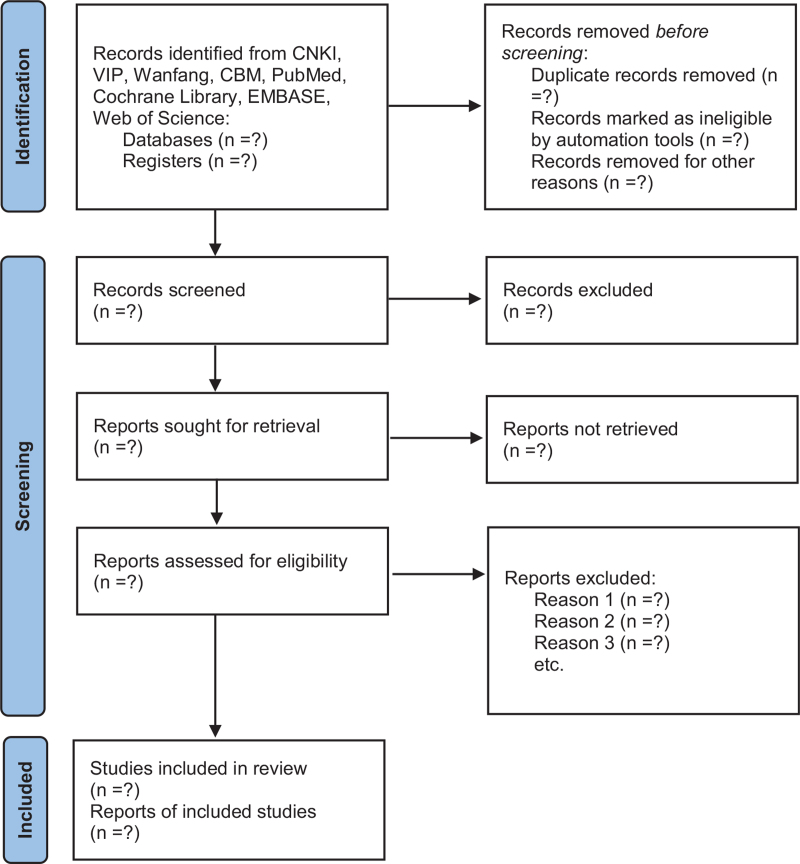
Flowchart of study selection procedures.

As a second step, 2 review team members (Fengyi Wang and Qinglin Li) will independently assess the studies’ eligibility by using the predefined inclusion and exclusion criteria. Besides, for the studies that meet the inclusion criteria, the whole article will be read by reviewers to ensure that the entire studies meet the criterion and prepare to extract relevant information. Any disagreements on whether to include a specific study or not will be resolved by discussion between the reviewers. The lacking information will be requested by contacting the writer of the original article. The flowchart of all study selection procedures is shown in Figure 1.

### 2.5 Data extraction

The information extracted from relevant literature by the 2 review team members will include study setting; study population and participant demographics and baseline characteristics; details of the intervention and control conditions; study methodology; outcomes and times of measurement; indicators of acceptability to users; suggested mechanisms of intervention action; information for the assessment of the risk of bias. Two reviewers will extract data independently; discrepancies will be identified and resolved through discussion (with a third author where necessary). We will request the missing data from the study authors.

### 2.6 Risk of bias Assessment

There will be 2 reviewers involved in the quality assessment process, and any major disagreements will be resolved by discussion to define the final set of included studies.

Two reviewers will independently assess the risk of bias in included studies by considering the following characteristics: randomization sequence generation, treatment allocation concealment, blinding method, completeness of outcome data, selective outcome reporting, and other sources of bias.

Besides, the Cochrane Collaboration's risk of bias assessment tool will be used to assess the individual included studies’ quality.

### 2.7 Data synthesis

We will use Review Manager 5.3 software to carry out the quantitative synthesis if the included studies are sufficiently homogeneous. Mean difference or standardized means difference will be used for continuous data. Risk ratio (RR) will be used for the analysis of dichotomous data. Both we will give a 95% confidence interval (CI). In the case of homogeneous data, if *I*^*2*^ ≤ 50%, *P* > .1, the fixed-effect model will be adopted for the meta-analysis. Otherwise, the sources of heterogeneity will be further analyzed. After excluding marked clinical heterogeneity, a random-effect model will be adopted to perform the meta-analysis. Sensitivity and bias risk analyses will also be performed.

#### 2.7.1 Analysis of subgroups

There are some planned subgroup analyses will be performed: The effectiveness rate of ACE alone for PFP and ACE combined with other therapies for PFP, different stage of PFP (eg, ≤3 months, >3 months), different treatment duration of ACE for PFP (eg, ≤1 months, >1 months), different material of suture (eg, Catgut suture, synthetic absorbable suture).

#### 2.7.2 Sensitivity analysis

Sensitivity analysis will be carried out to identify the robustness and stability of pooled outcome results by removing the low quality of studies.

#### 2.7.3 Reporting bias analysis

If there are ≥10 RCTs included, a funnel plot will be performed to evaluate the reporting bias.

### 2.8 Quality of evidence

We will assess the quality of evidence for outcomes by using the Grading of Recommendations Assessment, Development, and Evaluation (GRADE) system.^[12]^ The five items (limitations, inconsistency, indirectness, imprecision, and publication bias) will be used to assess each outcome.

### 2.9 Ethics and dissemination

There are no ethical approval requirements because this systematic review will be performed based on published studies. The findings of this systematic review will be published in a peer-reviewed journal.

## 3 Discussion

PFP is a rapid unilateral facial paralysis or paralysis of unknown etiology caused by severe impairments to facial function and appearance. TCM has been used for PFP in a range of medical practices and health interventions in China and other Asian countries for >2500 years. According to the theory of TCM, *pathogenic wind* is the cause of PFP. *Pathogenic wind* existing in which hierarchy of body is associated with the severity of clinical condition and prognosis. Superficial position of pathogenic wind leads to mild facial paralysis symptoms and mostly without sequelae commonly. On the contrary, deep position of *pathogenic wind* make facial paralysis seriously and cause sequelae more likely. After catgut or polydioxanone sutures implanted in muscles, the slowly resolve of suture in acupoints can achieve the aim of dispelling wind from muscles to skin. The pathogenesis of PFP is unclear, but it is generally considered to be related to infection, inflammation, and decreased immune function.^[13,14]^ From the view of modern medicine, ACE treats chronic disease by synthesizing and releasing of neurotransmitters, adjusting human immunity, inhibiting the release of inflammatory factors, reducing apoptosis, regulating cellular factors.^[15]^ In neurological disease, ACE can help to repair damaged neural circuits by lasting and gentle stimulation. ACE showed clinical potential for facial wrinkles and laxity for its stimulation effect is easy to accumulate.^[16]^ Besides, considering the cost of time, it is convenient for patients to seek treatment due to the frequency of ACE treatment is lower than Acupuncture and the time-to-treatment is less than Acupuncture.

The efficacy and safety of acupuncture treatment for PFP has been proved. As a long-term acupuncture treatment, the effectiveness and safety of ACE should be discussed. Some clinical trials using ACE to adjust facial injuries and disorders are published.^[17,18]^ However, there is still a lack of valid evidence supporting that ACE is effective for PFP. Consequently, the purpose of this systematic review is to identify available RCTs of PFP that using ACE to offer persuasive evidence of efficacy and safety for clinical practitioners, scientific researchers, and even general patients.

## Author contributions

**Conceptualization:** Fengyi Wang, Qinglin Li, Qiaoyun Yu, Junquan Liang, Yunxiang Xu, Guizhen Chen.

**Data curation:** Junquan Liang, Guizhen Chen.

**Formal analysis:** Fengyi Wang, Qinglin Li.

**Funding acquisition:** Yunxiang Xu, Guizhen Chen.

**Methodology:** Fengyi Wang, Qinglin Li, Guizhen Chen.

**Project administration:** Yunxiang Xu, Guizhen Chen, FY Wang.

**Resources:** Fengyi Wang, Guizhen Chen.

**Software:** Fengyi Wang, Qinglin Li.

**Visualization**: Fengyi Wang, Qiaoyun Yu.

**Writing – original draft:** Fengyi Wang, Qinglin Li, Qiaoyun Yu.

**Writing – review & editing:** Junquan Liang, Yunxiang Xu, Guizhen Chen.
